# Surface-Conjugated Galactose on Electrospun Polycaprolactone Nanofibers: An Innovative Scaffold for Uterine Tissue Engineering

**DOI:** 10.1021/acsomega.3c10445

**Published:** 2024-08-01

**Authors:** Srividya Hanuman, Harish Kumar B, K. Sreedhara Ranganath Pai, Manasa Nune

**Affiliations:** † Manipal Institute of Regenerative Medicine, Manipal Academy of Higher Education, Manipal, Karnataka 576104, India; ‡ Department of Pharmacology, Manipal College of Pharmaceutical Sciences, Manipal Academy of Higher Education, Manipal, Karnataka 576104, India

## Abstract

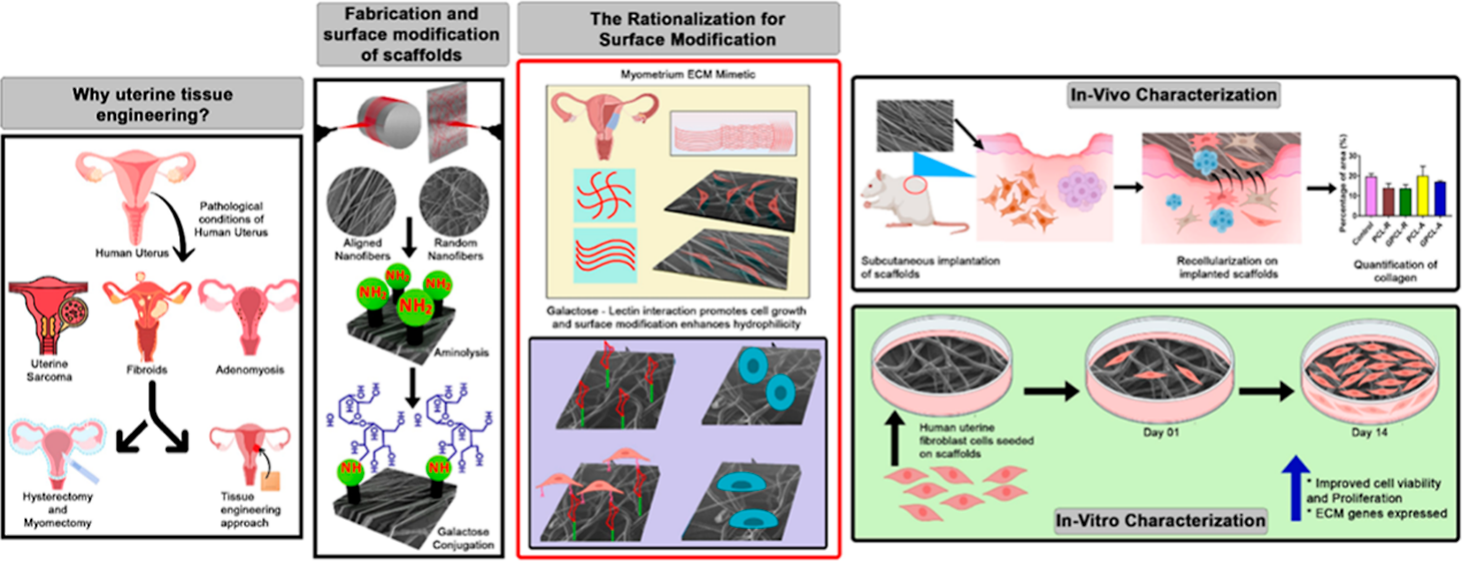

The uterus, a vital organ in the female reproductive system, nurtures and supports developing embryos until maturity. This study focuses on addressing uterine related problems by creating a nanofibrous scaffold to regenerate uterine myometrial tissue, closely resembling the native extracellular matrix (ECM) for enhanced efficacy. To achieve this, we utilized polycaprolactone (PCL) as a biomaterial and employed an electrospinning technique to generate PCL nanofibers in both random and aligned orientations. Due to the inherent hydrophobic nature of PCL nanofibers, a two-step wet chemistry surface modification technique is used, involving the conjugation of galactose onto them. Galactose, a lectin-binding sugar, was chosen to enhance the scaffold’s hydrophilicity, thereby improving cell adhesion and fostering l-selectin-based interactions between the scaffold and uterine cells. These interactions, in turn, activated uterine fibroblasts, leading to ECM remodeling. The optimized electrospinning process successfully generated random and aligned nanofibers. Subsequent surface modification was carried out, and the modified scaffold was subjected to various physicochemical characterization, such as the ninhydrin assay, enzyme-linked lectin assay techniques that revealed successful galactose conjugation, and mechanical characterization to assess any changes in material bulk properties resulting from the modification. The tensile strength of random galactose-modified PCL fibers reached 0.041 ± 0.01 MPa, outperforming random unmodified PCL fibers (0.026 ± 0.01 MPa), aligned unmodified PCL fibers (0.011 ± 0.001 MPa), and aligned modified PCL fibers (0.016 ± 0.002 MPa). Cytocompatibility studies with human uterine fibroblast cells showed enhanced viability and proliferation on the modified scaffolds. Initial pilot studies were attempted in the current study involving subcutaneous implantation in the dorsal area of Wistar rats to assess biocompatibility and tissue response before proceeding to intrauterine implantation indicated that the modification did not induce adverse inflammation in vivo. In conclusion, our study introduces a surface-modified PCL nanofibrous material for myometrial tissue engineering, offering promise in addressing myometrial damage and advancing uterine health and reproductive well-being.

## 1 Introduction

The mammalian uterus is a complex female reproductive organ that plays a vital role in the various stages of reproduction. It is composed of three distinct layers: the innermost layer, known as the endometrium; the middle layer, referred to as the myometrium; and the outermost layer, called the perimetrium.^1^ This study focuses on the myometrium, a specialized layer primarily composed of smooth muscle cells within the uterus. The myometrium plays a crucial role in maintaining pregnancy and initiating childbirth. The myometrium undergoes a complex and dynamic physiological process known as uterine contractility, which is observed in both nonpregnant and pregnant women phases across the menstrual cycle and is placed in the group of spontaneously active and readily excitable muscle tissue.^2^ Hormones such as estradiol, oxytocin, and prostaglandins exert a substantial influence on myometrial function, contributing to its growth, contraction, and effective functioning.^3^

Various complex and intricate interplays of molecules and interactions take place within the uterus. Among these biomolecules, carbohydrates hold particular importance, serving crucial functions throughout different stages of reproduction. The mammalian uterus consists of a high concentration of glycoconjugates, primarily engaging implanted embryos, fetuses, and protecting them from potential pathogens that may enter the uterine environment.^4^ Studies have confirmed that the surface of the uterus expresses various sugar epitopes that are detected by lectin proteins. These epitopes are regulated during various stages of pregnancy, including preimplantation and postimplantation. It has been established that uterine and trophoblast cells express both sugar-binding proteins and cell surface glycoconjugates. For successful implantation to occur, the uterine lining must be receptive. Carbohydrate recognition has been observed to play a crucial role in controlling the implantation of the human embryo during the initial stages.^5,6^ In another study, it was shown that trophoblasts have galactose present in them, which is recognized by them human uterus for invasion and implantation.^7^

The uterus, while possessing remarkable regenerative abilities, is susceptible to various diseases and conditions. Notable among these are fibroids, also known as leiomyomas, which are noncancerous growths that develop within the uterine wall, potentially causing discomfort and complications.^8^ Another condition is adenomyosis, where endometrial glands migrate into the myometrial region of the uterus, resulting in an enlarged uterus.^9^ Furthermore, certain medical procedures, like cesarean deliveries and uterine surgeries, although often necessary, can impact the uterus. These interventions may lead to complications in the form of scarring. These scars have the potential to disrupt the normal structure of the myometrium, which can give rise to issues such as abnormal placenta placement and uterine rupture.^10^

Traditional approaches such as allografts and transplants, while effective in some cases, are not without their significant drawbacks, including the risk of rejection, infections, and complex postsurgical complications. In light of these challenges, it is important to consider alternative approaches. One such approach involves tissue engineering using principles to create new scaffolds tailored to specific tissues is essential, which has been explored in this study to design a patch to treat the region of scar or wound in the myometrium. This includes designing scaffolds for the uterus, which is a promising way to tackle important medical issues related to the uterus, pregnancy, and childbirth.^11^ In tissue engineering, biomaterials play an important role by providing a framework similar to the extracellular matrix (ECM) structure.^12^ In order to mimic the ECM fibrous structure of tissues, the electrospinning technique is used to create nanofibrous scaffolds.^13^ Various polymers have been used for electrospinning nanofibers, including both natural and synthetic polymers. However, it is more convenient to process synthetic polymers through electrospinning, which aids in better control of the nanofiber morphology compared to natural polymers. Natural polymers are primarily water-soluble, which poses challenges in directly converting them to nanofibers due to their inherent instability. Additionally, they are susceptible to harsh processing conditions because of their mechanical weakness. In contrast, synthetic polymers offer greater versatility for specific biological functions while exhibiting desirable properties such as a cost-effective and easily scalable approach to scaffold development, ensuring excellent mechanical strength and surface integrity.^14,15^

Polycaprolactone (PCL), an aliphatic polyester, was utilized as a biomaterial in this study. It has a melting point of 55 °C and remarkable load-bearing mechanical characteristics. It is ideal for soft tissue engineering, especially for myometrium.^16^ Further, we have compared two different types of nanofibers: randomly oriented and aligned nanofibers. A recent study by Miki et al. examined the orientation of decellularized uterine scaffolds (DUS) in rats revealed that scaffold orientation significantly influences uterine tissue regeneration. Incorrectly oriented DUS led to aberrant tissue topology.^17^ Building on this insight, we conducted a comparative analysis of random and aligned nanofibers for uterine tissue regeneration, considering the myometrium’s complex layers, which are oriented longitudinal, crisscross, and circular.^18^

PCL is preferred for regeneration, but it has a hydrophobic nature. To address the issue of the hydrophobic nature of PCL, in this study, PCL fibers are modified using chemical methods in order to establish improved cell-scaffold contact and integration. We have used amine groups to conjugate galactose on the surface of PCL nanofibers. This design is based on a comprehensive understanding of the role of carbohydrates, particularly galactose. In this study, the novelty lies in the development of galactose-conjugated PCL nanofibrous scaffolds tailored to enhance myometrial regeneration, concurrently establishing a biomimetic environment and simultaneously creating a biomimetic environment. The rationale behind the conjugation of galactose is that by modifying polymeric scaffolds with carbohydrate molecules like galactose, we aim to replicate the natural interactions that occur within the uterine wall and blastocyst. The addition of galactose causes the l-selectin-based interaction of uterine cells with fibers. The ECM is remodeled as a result of uterine fibroblast activation brought on by l-selectin–galactose interaction.^19^ This modification enhances the scaffold’s ability to encourage cell attachment, growth, and tissue repair. The initial step of aminolysis involves the addition of amino groups to PCL fibers by breaking ester bonds present on the PCL,^20^ and the second step is the addition of galactose, where galactose is added using lactose, where anomeric carbon of the glucose will covalently attach to the surface and galactose is exposed on the surface.^21^ The goal of this surface modification is to improve the hydrophilicity of the surface of the polymer without altering its mechanical strength. The addition of galactose is used to increase the hydrophilicity of PCL which further improves cell adhesion on fibers. This patch presents a remedy for the efficient regeneration and recovery of uterine myometrium wounds, with the potential to transform the approach to managing such injuries related to uterine tissue.

## 2 Materials and Methods

### 2.1 PCL Solution

Poly(ε-caprolactone) (Mn = 80,000) pellets were obtained from Sigma-Aldrich. The solvents chloroform and methanol to dissolve PCL were obtained from Himedia Co. For the preparation of the 10% (w/v) solution, the first PCL pellets were added to chloroform and stirred for 15 min. Later, to the same solution, methanol was added in the ratio of 4:1 and kept for 12 h stirring at room temperature until the pellets were dissolved completely.^16^

### 2.2 Electrospinning

Nanofibers were produced using an electrospinning unit (Model-HO-NFES-040) with a set of optimized parameters. The electrospinning parameters were optimized and described in a prior study by our group.^16^ A 10 mL syringe containing polymer was set, and a voltage of 18 kV was applied. A needle with a gauge of 24G was used, and a constant distance of 20 cm was maintained between the needle tip and the collector, while the flow rate was accurately held at 0.002 mL/min. A rotating mandrel with a speed of 2000 rpm was used for aligned fibers. The electrospinning was carried out for 24 h to obtain a single mat of random or aligned fibers. The random fibers and aligned fibers collected on the stationary and rotating mandrels were stored in a vacuum desiccator for further characterization.

### 2.3 Surface Modification

The surface modification of PCL nanofibers was done by conjugating the surface of the nanofibers with galactose in two steps.

#### 2.3.1 Step-1 Aminolysis

PCL nanofibrous mats obtained are cut into pieces measuring 1 × 1 cm. To eliminate oil or other dirt on scaffolds, it was immersed in an alcohol–water (1:1, v/v) solution, washed with deionized water, and dried. Fibers were then immersed in a 10% 1,6-hexane diamine/2-propanol solution overnight at 37 °C, rinsed with deionized water to remove any unattached or excess 1,6-hexane diamine, and dried.^16^

#### 2.3.2 Step-2 Galactose Grafting

To conjugate galactose on the aminated scaffolds, the aminated discs were soaked in 100 mL of citrate buffer solution overnight containing 1.88 g of sodium cyanoborohydride (NaBH3CN) and 21.61 g of galactose, with a pH of 6.1, following the neo glycosylation protocol from ref (22).

### 2.4 SEM Analysis

Morphology and post surface modification structure of electrospun fibers were analyzed using scanning electron microscopy (SEM, Carl Zeiss Ultra 55, CeNSE, IISc Bangalore). Prior to analysis, samples were desiccated for 24 h in a desiccator to remove any solvents present and then gold sputter-coated to apply a conductive layer before mounting. Analysis was conducted at magnifications of 75K, 25K, and 5K X at an accelerating voltage of 5 kV. SEM images were utilized for fiber diameter and orientation analyses using ImageJ software.

### 2.5 Ninhydrin Assay

Post-aminolysis, a ninhydrin assay was performed to quantify the presence of amine groups. Ninhydrin reagent (1 M) prepared in 10 mL of ethanol was added to the scaffold discs. Scaffolds were incubated in 100 μL of ninhydrin solution in a hot water bath at 70 °C for 15 min. Subsequently, the tubes were allowed to cool to room temperature. To dissolve the scaffolds, 500 μL of chloroform and isopropyl alcohol were added to the tubes. From this solution, 100 μL was transferred to 96-well plates, and the intensity was measured at a wavelength of 562 nm using a spectrophotometer [PerkinElmer (Ensight) multimode plate reader HH34000000].

### 2.6 Enzyme Linked Lectin Assay (ELLA)

To quantitatively and qualitatively assess the amount of galactose conjugated on the nanofiber’s surface, two types of ELLA assays were conducted. In the first assay, a lectin with FITC fluorescence was used (FITC-ELLA). In the second assay, a lectin conjugated with horseradish peroxidase (HRP) was employed. For the FITC-ELLA assay, PCL and galactose-conjugated PCL samples were suspended in a phosphate-buffered saline (PBS) solution containing FITC-conjugated lectin from *Arachis hypogaea* (peanut lectin) (Sigma-Aldrich L7381) at a concentration of 40 μg/mL. The samples were then stirred in the dark for 2 h. After this incubation, they were washed with PBS. Subsequently, the samples were examined for their fluorescence using a fluorescence microscope (Nikon Eclipse-TE2000-U).

The HRP-ELLA assay involved treating PCL and galactose-conjugated PCL samples with a 2% BSA solution in PBS (100 μL) and shaking them at 5 °C for 14 h. Subsequently, the samples were incubated at room temperature with a solution of peanut lectin conjugated to HRP (Sigma-Aldrich, L7759) (0.01 mg/mL, 200 μL) in PBS (200 μL) for 2 h with shaking. After incubation, excess unbound lectin was removed by thorough washing with PBS. Next, the samples were treated with a solution of OPD (*o*-phenylenediamine dihydrochloride) (SIGMAFASTM OPD Sigma-Aldrich, catalog no. P9187) for 1 h. The absorbance of a 200 μL aliquot of this solution was then measured at 450 nm using a spectrophotometer [PerkinElmer (Ensight) multimode plate reader HH34000000].

### 2.7 Contact Angle Analysis

The hydrophobicity and hydrophilicity of the nanofiber surface were confirmed with a contact angle using a goniometer. Each scaffold (*n* = 3) was considered for the study, and using a water droplet, the angle between the water droplet and surface was used to study the surface energy of the scaffolds at room temperature 23–25 °C.

### 2.8 Mechanical Characterization

The mechanical characteristics of unmodified and modified PCL nanofibrous scaffolds in both random and aligned configurations were evaluated using a Shimadzu universal texture analyzer (EZ-SX) device, manufactured by Shimadzu Corporation, Japan. Electrospun mats were trimmed to produce samples measuring approximately 100 mm in length and 20 mm in width. These samples were securely clamped at both ends and subjected to a constant stretching rate of 10 mm/min until they reached the point of fracture. The collected data was subsequently transformed into stress–strain curves, and tensile strength as well as the percentage of elongation at break were determined based on the sample’s width and thickness. The results are presented as the mean value ± the standard deviation based on three separate measurements.

### 2.9 Water Absorption and Degradation Assays

Nanofiber scaffolds, cut into 1 cm^2^ pieces, were immersed in PBS (pH = 7.4) and incubated in vitro at 37 °C for 7, 14, and 21 days. At these intervals, water uptake and degradation were assessed. Water uptake was determined by measuring the wet weight of the scaffolds after blotting excess surface water. Subsequently, the scaffolds were washed, dried for 24 h at room temperature, and weighed to assess degradation. Additionally, morphological changes were observed using SEM analysis.^23^

### 2.10 Cell Culture Studies

Human uterine fibroblast cells (HUF) (PCS-460-010) were maintained using fibroblast basal medium (ATCC-PCS-201-030) supplemented with the Fibroblast Growth Kit-low Serum—(ATCC PCS-201-0410) and 1% penicillin–streptomycin. The cultures were incubated at 37 °C in 5% carbon dioxide. Subsequently, confluent HUF was seeded onto scaffolds for further investigation.

### 2.11 MTT Assay

The scaffolds were cut to fit the size of a 96 well plate and then sterilized under UV in the laminar hood for 24 h before cell seeding. HUF were seeded onto the scaffolds at a density of 5000 cells per well. Scaffolds were cut into 5 mm diameters each and placed in the 96 well plate, and tissue culture polystyrene (TCPS) was kept as a control. The plate was then placed in a CO_2_ incubator at 37 °C. Readings were taken at three-time intervals: 1, 7, and 14 days after culturing. The plate was incubated for 3–4 h with an MTT reagent (0.5 mg/mL). Afterward, 100 μL of DMSO reagent was added to each well and left for 1 h to dissolve the formazan crystals, resulting in a color change. Finally, the absorbance of the formazan solution was measured at 570 nm using a spectrophotometer [PerkinElmer (Ensight) multimode plate reader HH34000000].

### 2.12 Live/Dead Assay

Cell viability of the seeded cells on the scaffolds was evaluated at two different time points, day 1 and day 3, using a live/dead assay kit (L3224). To prepare the live/dead reagent, a stock solution was created by combining 4 μL of EthD-1 and 1 μL of Calcein-AM in 2 mL of PBS. Subsequently, 100–150 μL of the live/dead reagent was added to the scaffolds and incubated at room temperature for 1 h. The cells were then examined by using a fluorescence microscope (Nikon Eclipse-TE2000-U).

### 2.13 Immunofluorescence Assay

Cells were fixed with 4% paraformaldehyde for 1 h at room temperature, after fixing, additional PBS washes were performed, followed by permeabilization using 0.5% Triton X-100 for 30 min at room temperature. The cells were then blocked with 5% BSA for 1 h at room temperature. The cells were later incubated with the appropriate dilution of primary antibody-Versican (1:100 dilution, NBP2-22408 Novus Biologicals) overnight at 4 °C. Washes were performed gently, and secondary antibody Rabbit anti-Mouse IgG1 fluorescein (NBP1-73636 Novus Biologicals) was added at a final concentration of a 1:1000 for 1 h at room temperature. It was counterstained with rhodamine-phalloidin stain (R415, Invitrogen) for 45 min and washed with PBS. The cells were stained with 1:1000 diluted DAPI solution, and they were visualized under the fluorescence microscope (NikonTE2000U) with filters.

### 2.14 Gene Expression Analysis

Total RNA is extracted from the HUF cultured on the scaffolds for 7 and 14 days using the standard Trizol RNA (RNA iso Takara) isolation protocol. The obtained RNA is later subjected to cDNA synthesis using the kit (RDRT Sigma-Aldrich ReadyScript cDNA Synthesis Mix) according to the manufacturer’s instruction. Then, the mRNA expression is carried out by real-time PCR using the SYBR Green master mix (BioRad iTaq Universal SYBR Green Supermix). The experimental procedure was conducted with a total volume of 10 μL, comprising 0.5 μL of each primer, as listed in Table 1, 5 μL of SYBR green master mix, 3.5 μL of diethylpyrocarbonate-treated water, and 0.5 μL of cDNA template. Subsequently, the samples were subjected to 100 cycles using the Qiagen Rotar Q series machine, and the obtained results were analyzed using the accompanying software, Rotorgene Qiagen software for the instrument.

**Table 1:** Primer Sequences Used for Gene Expression Analysis

primer	forward sequence	reverse sequence
GAPDH	CCATGGGGAAGGTGAAGGTC	TGGAATTTGCCATGGGTGGA
galectin 3	CACCTGCACCTGGAGTCTAC	TGTTATCAGCATGCGAGGCA
versican	GACCAGTGCGATTACGGGT	GCAGCGATCAGGTCGTTTA
laminin	TCAGTTTCTTAGCCCTGTGC	CGATACAGTAGGGTTCGGGC
collagen I	TGACGAGACCAAGAACTGCC	GCACCATCATTTCCACGAGC
collagen III	CGCCCTCCTAATGGTCAAGG	CCAGGGTCACCATTTCTCCC

### 2.15 Animal Studies

Wistar rats (*Rattus norvegicus*) were used for in vivo experiments. All animals were provided with care in strict adherence to the guidelines established by the Kasturba Medical College, Manipal, MAHE, for Animal Care. Furthermore, the Institute’s Ethical Review Committee granted approval for the experimental protocols under reference number IAEC/KMC/76/2022. For each group, 3 rats were assigned randomly. Each animal weighed between 250 and 300 g. Intraperitoneal injections of ketamine and xylazine were administered to anesthetize animals based on their weight. The dorsal area of the animals was shaved and sterilized with 70% ethanol. Using a sterile surgical blade, an incision of about 1 cm was made on the dorsal lobes of animals. A subcutaneous pouch was created on the incision. Scaffolds were UV sterilized before using for implantation, and an implant was inserted into each pocket. Upon implantation of the polymer into the pouch, the cut was sutured. The sutures were removed 7 days after surgery. After 3 weeks, the tissue surrounding the implant was excised to study and understand the inflammatory response by using hematoxylin and eosin (H&E) staining and Masson’s trichome staining.

### 2.16 H&E Staining

Haematoxylin stain’s acidic part, stains mainly the nucleus, while eosin acts as an acidic stain and binds the basic part, i.e., the cytoplasm. The samples were fixed with methanol for 30 min, and after drying in air, different ranges of alcohols such as 100, 70, and 40% are added for a few seconds each for hydration. The samples were further stained in hematoxylin for 20 min and washed with 1% acetic acid for 10 min until the nuclei appeared blue; eosin was added for 30 s, washed with distilled water, and further treated with a different range of alcohol for dehydration.

### 2.17 Masson’s Trichome Staining

Tissue samples were fixed in 4% paraformaldehyde at 4 °C for 24 h and then paraffin embedded. After fixation, slides were stained with Weigert’s iron hematoxylin for 10–15 min, which stained the nuclei blue–black, and rinsed in distilled water. Slides were immersed in Biebrich scarlet acid fuchsin for 5–10 min, which stained the muscle fibers and cytoplasm red, and then rinsed. Tissue sections were differentiated with phosphomolybdic–phosphotungstic acid for a few minutes, which removes excess stain from collagen, and rinsed. Slides were finally immersed in aniline blue for 5–10 min, which stained collagen fibers blue green. Stained sections were dehydrated in alcohol (70, 95, and 100% ethanol), cleared in xylene, and mounted using Permount. After drying, Masson’s trichrome-stained tissue sections were ready for microscopic examination, aiding collagen, nuclei, and muscle fiber visualization for tissue analysis. Subsequently, the image intensities were calculated by using ImageJ software.

### 2.18 Statistical Analysis

The data were collected with replication schemes, and mean values were calculated for each set. Statistical analysis was performed using GraphPad Prism software with ANOVA. Significance levels were represented as follows: *p < 0.05; **p < 0.01; ***p < 0.001; and ****p < 0.0001.

## 3 Results and Discussion

### 3.1 SEM Analysis of Random and Aligned Nanofibers

**Figure 1:**
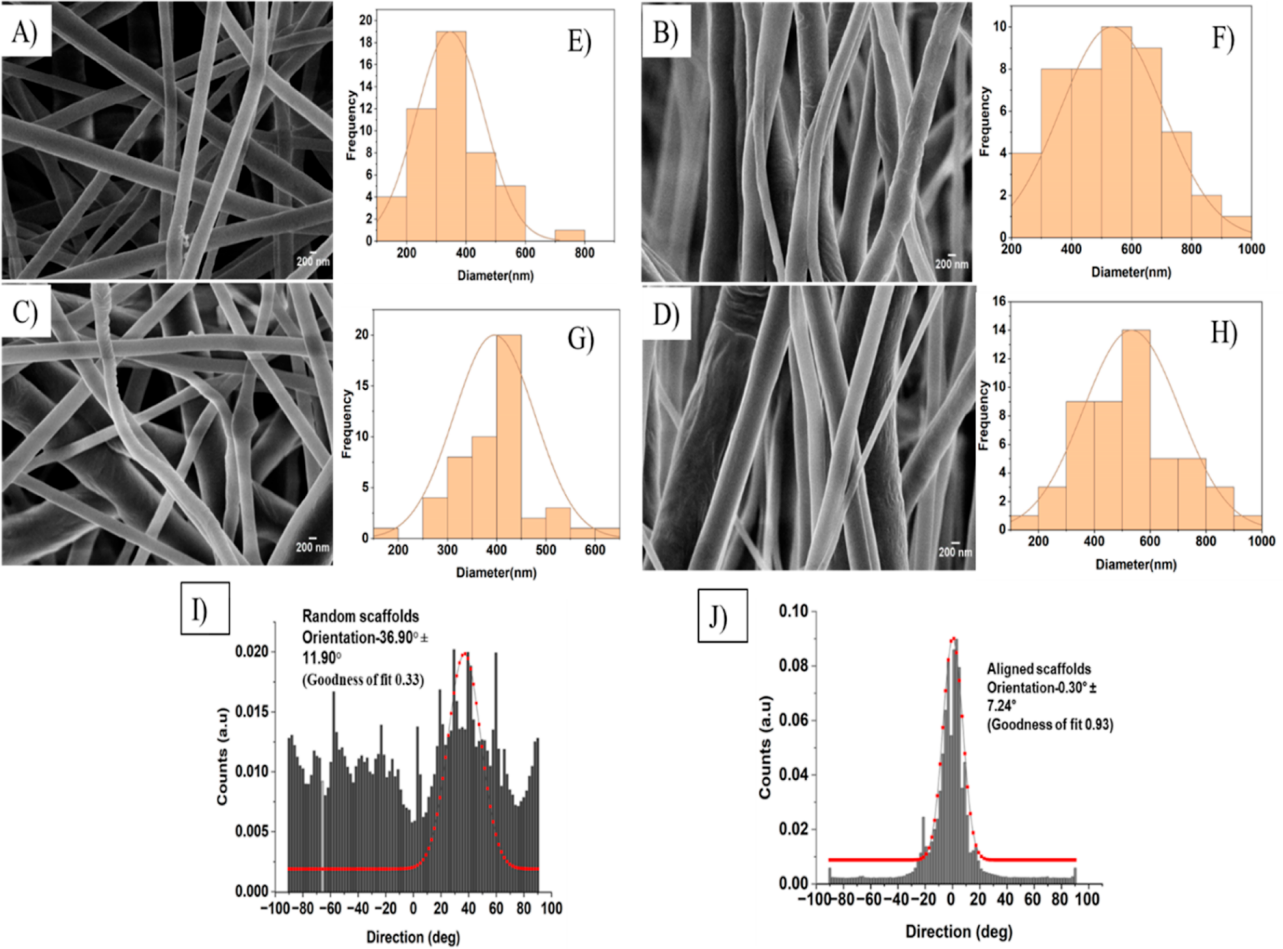
SEM images of (A) PCL random (PCL-R), (B) PCL aligned (PCL-A), (C) GPCL-R, and (D) GPCL-A. (E–H) Histograms of fiber diameter (minimum 100 fibers were measured) (E) PCL-R, (F) PCL-A, (G) GPCL-R, and (H) GPCL-A. (I,J) Histograms showing the alignment of nanofibers.

In this study, the electrospinning technique was utilized to fabricate PCL nanofibers to replicate the intricate ECM present in the uterus myometrium. This method yielded nanofibers that closely mimic the tissue’s ECM structure. Subsequently, the PCL nanofibers were subjected to surface modification with galactose. The primary focus of the SEM analysis was to investigate four distinct characteristics: morphology, diameter, porosity, and orientation. Beginning with morphology, in Figure 1 the nanofibers displayed a smooth, bead-free structure in both random and aligned configurations. Post modification, no significant roughness was observed. Similarly to the previous study, post maltose conjugation using a similar technique made the nanofiber’s surface smooth.^24^ The treatment time plays a crucial role, as the morphology will be damaged and the fibers will break if they are treated for a longer time.^25^ Regarding diameter, the nanofiber measurements fell within the nanorange, i.e., 1–1000 nm. The diameter of random PCL fibers was found to be 360.9 ± 151.3 nm, and aligned PCL fibers had 570.7 ± 219.53 nm. The diameter of the nanofibers is affected by the type of collector; in aligned nanofibers, in order to obtain less deviation in alignment angle, the rotating mandrel speed was set to 2000 rpm, which resulted in the diameter variation, whereas random fibers resulted in a consistent nanometer range.^26^ Notably, after modification, the morphology and diameter of the fibers changed slightly, random fiber diameter was 395.6 ± 81.4 nm, and aligned fibers diameter was 591.4 ± 304.1 nm.

Through image analysis software (ImageJ), the porosity of both aligned and random fibers was quantified. As shown in Table 2, it is noteworthy that aligned fibers exhibited lower porosity compared with random fibers. Specifically, random fibers demonstrated a porosity of 13.8%, while aligned fibers displayed a porosity of 4.5%. After surface modification, galactose PCL random (GPCL-R) has 11.6% and galactose PCL aligned (GPCL-A) has 7% of porosity, respectively. Aligned fibers are densely packed when compared to random fibers; therefore, their porosity is reduced.^27^Figure 1I,J shows that the angle of alignment for the fibers is approximately 0.30 ± 7.24° for aligned fibers, and for random fibers, the alignment is not in a single direction, but most of the fibers were around 36.90 ± 11.90°. The above results indicate that aminolysis and galactose treatment had no remarkable effect on morphology, whereas diameter increased slightly when compared to unmodified PCL. This slight increase in diameter may be attributed to the tendency of nanofibers to form fused structures during surface modification, which consequently led to a slight increase in diameter and porosity.^28^ These findings align with similar studies; for instance, in our previous study^24^ we reported that aminolysis and sugar conjugation did not affect the surface morphology of fibers, and the diameter did not change. In addition, in a study by Amoures de Sousa et al., no alteration was found in the morphology of PCL nanofibers following the modification.^68^

**Table 2:** Porosity of PCL-R, GPCL-R, PCL-A, and GPCL-A Nanofibers

sample	magnification (X)	total area of image (μm)	total pore area (μm)	average pore size (μm)	porosity percentage (%)
PCL-R	5000	1222	169.1	0.014	13.8
GPCL-R	5000	1373	159.7	0.011	11.6
PCL-A	5000	1359	61.8	0.004	4.5
GPCL-A	5000	1442	102.3	0.006	7.1

### 3.2 Ninhydrin Assay

The process of aminolysis involves breaking ester bonds present on PCL, leading to the generation of amide bonds. This study utilizes 1,6-hexanediamine to perform this reaction on PCL. This reaction results in one amino group, reacting with the –COO– group to establish a covalent –CONH– bond, while the second amino group remains unreacted and free. This available free amino group is subsequently used to conjugate galactose; this reaction is an intermediate step for galactose conjugation.^28−31^

**Figure 2:**
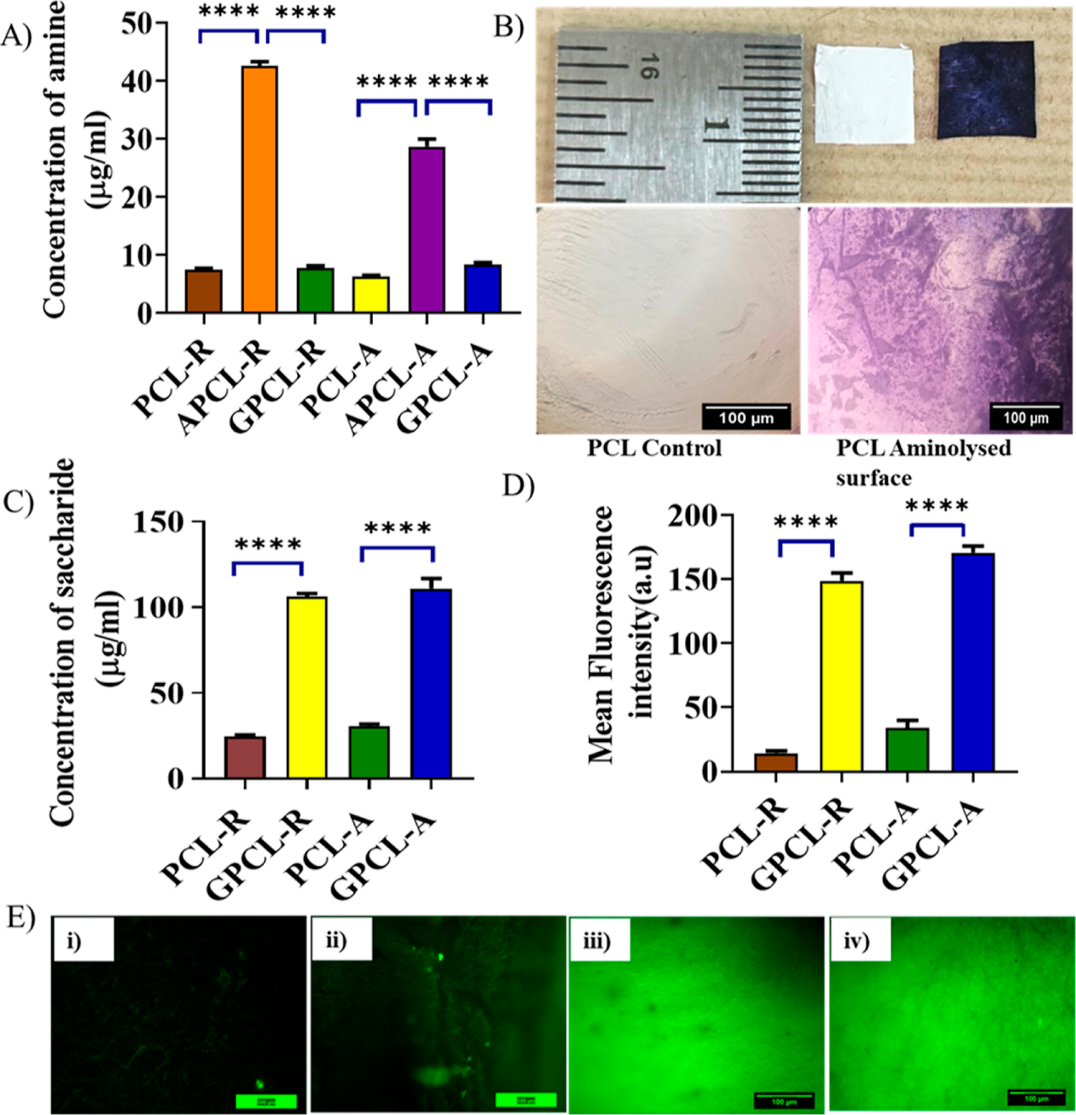
(A) Quantification of amine groups using the ninhydrin assay on PCL-R, APCL-R, GPCL-R, PCL-A, APCL-A, and GPCL-A (***p < 0.001). (B) Macroscopic picture of ninhydrin reaction color transition on the surface-modified scaffold. Scale bar = 100 μm. (C) ELLA-HRP-conjugated assay quantification of the galactose moiety (****p < 0.0001). (D) ELLA-FITC conjugated assay-quantitative analysis of the galactose moiety (****p < 0.0001). (E) Fluorescent images of the ELLA-FITC assay on (i) PCL-R, (ii) PCL-A, (iii) GPCL-R, and (iv) GPCL-A nanofibers.

The confirmation test for the presence of a free amino group on the surface was achieved through the ninhydrin test. The ninhydrin (triketohydrindene hydrate) test, a simple and sensitive assay, works on the basis of reacting with the primary amino group to produce a colored product known as diketohydrindylidene-diketohydrindamine (Ruhemann’s Purple), as shown in Figure 2B.^20^ As represented in Figure 2A, the amine concentration of the samples was calculated using the standard curve obtained using 1,6 hexanediamine. Aminolyzed random fibers showed 42.6 ± 0.6 μg/mL and aligned fibers showed 28.6 ± 1.2 μg/mL of amine concentration. Since random fibers are more porous, and aminolysis occurs in depth in the *Z* direction; the amount of amine groups is higher on random fibers when compared to aligned fibers.^31^ On GPCL-R fibers, it decreased to 7.7 ± 0.4 μg/mL and in GPCL-A, it decreased to 8.4 ± 0.4 μg/mL, which is similar to unmodified random and aligned PCL fibers, which had 7.2 ± 0.2 and 6.3 ± 0.2 μg/mL of amine concentration, respectively. A similar decrease in the percentage of amine groups after conjugation with galactose and maltose was also observed in a few other studies.^21,22^ The NH_2_ group participates in the conjugation of galactose; therefore, free amine groups are not available to react with the ninhydrin reagent.^32^

### 3.3 ELLA

Carbohydrates can only perform their biochemical role if they are exposed to the appropriate receptors.^33^ Galactose is conjugated on the surface by using lactose sugar. Lactose sugar has two components, glucose and galactose. Glucose attaches to free amine groups, and galactose is present on top. Therefore, to confirm the galactose moiety on the top of the surface, an ELLA assay is performed. Both quantitatively and qualitatively, the galactose moiety present is calculated using HRP and FITC-conjugated peanut lectin from *A. hypogaea*. To quantitatively measure the quantity of galactose, we used a standard curve of lectin. As shown in Figure 2C, the galactose content is determined to be 106.2 ± 0.12 μg/mL on random nanofibers and 110.9 ± 0.4 μg/mL on aligned nanofibers. To qualitatively see the spread of galactose on the surface, the FITC-conjugated ELLA assay was done. We can clearly differentiate between galactose-conjugated intensity in Figure 2E(iii,iv) on the scaffolds conjugated with galactose compared to the unmodified scaffolds in Figure 2E(i,ii), and the intensity of the fluorescence is calculated and depicted in graph Figure 2D. This confirms the presence of a galactose moiety on top of the fibers surface.

### 3.4 Contact Angle Analysis

**Figure 3:**
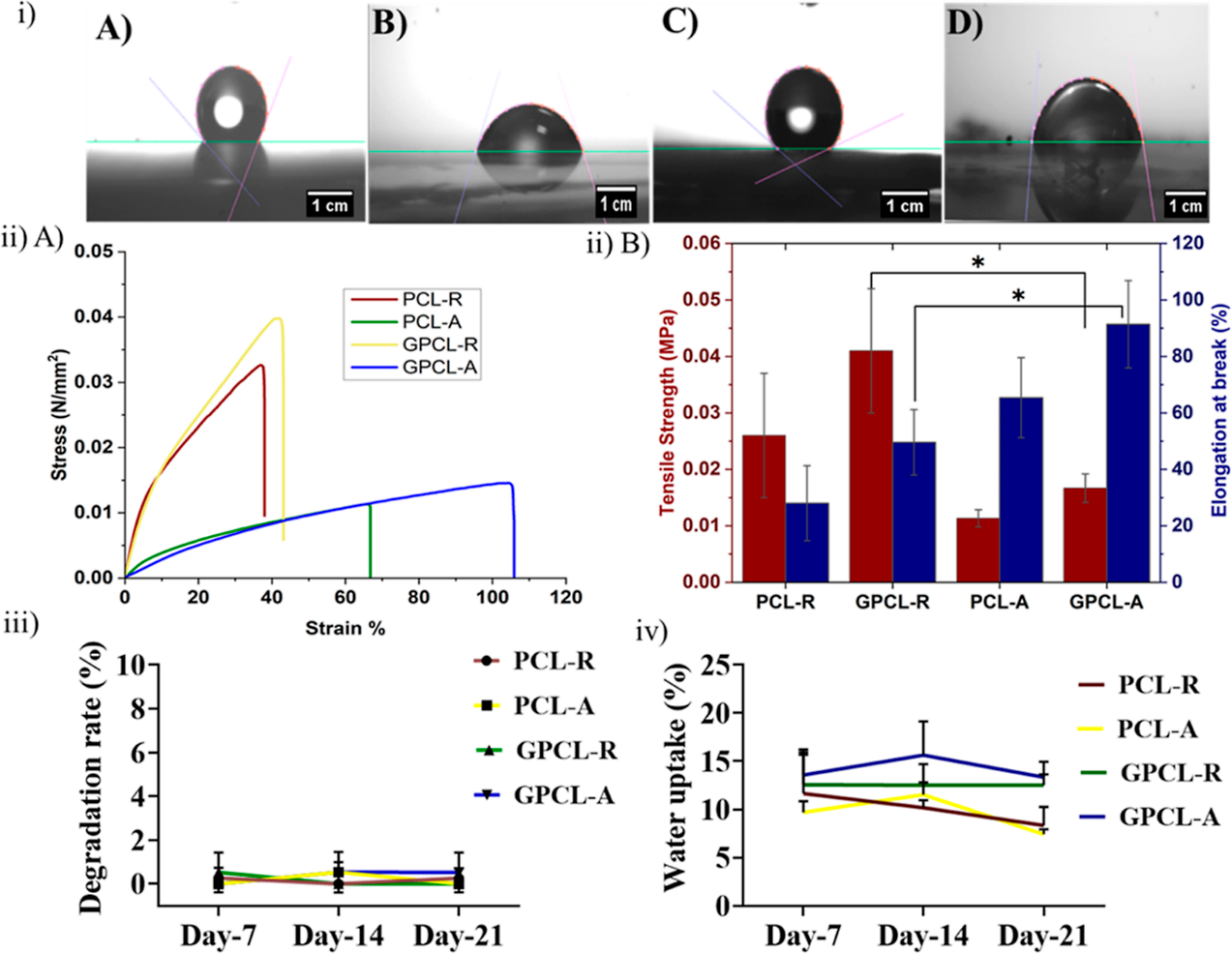
(i) Contact angle of (A) PCL-R, (B) GPCL-R, and (C) PCL-A, (D) GPCL-A nanofibers. (ii) Mechanical properties of unmodified PCL-R, PCL-A, and modified GPCL-R and GPCL-A scaffolds. (A) Representative stress–strain curves. (B) Tensile strength and elongation at break. Data are presented as average ± standard deviation (*n* = 3). (iii) Degradation rate of nanofibers: (A) PCL-R, (B) PCL-A, (C) GPCL-R, (D) GPCL-A, and (iv) Water uptake kinetics of nanofibers (A) PCL-R, (B) PCL-A, (C) GPCL-R, and (D) GPCL-A.

Surface wettability, referred to as hydrophobicity or hydrophilicity, stands as a critical factor influencing various cellular behaviors. The degree of wettability of the scaffolds was determined through water contact angle measurements, a reliable parameter that measures how readily water droplets spread on the nanofibrous surface.^34^ The results of the contact angle shown in Table 3 show that unmodified PCL-R fibers exhibited hydrophobic traits, with a left contact angle of 134.94 ± 3.31° and right contact angle of 135.52 ± 3.09°. On the other hand, galactose-conjugated PCL surfaces display reduced contact angles, with a left angle of 77.85 ± 8.0° and a right angle of 78.14 ± 3.9°. Similarly, aligned PCL fibers show a right angle of 127.63 ± 3.09° and a left angle of 128.4 ± 3.2°, while galactose-conjugated aligned PCL surfaces exhibit even further reduction to 64.63 ± 8.0 and 65.14 ± 3.9° of left and right angles, respectively. The surface modification involving galactose results in the introduction of hydroxyl groups and an increase in surface energy, consequently enhancing hydrophilicity.^35^ Additionally, it was observed in Figure 3iB,D that water droplets spread more rapidly on galactose-grafted PCL surfaces and were quickly absorbed upon contact. This phenomenon promotes the attachment of negatively charged cells to the surface of the modified PCL fibers.^36^ Furthermore, scaffold alignment revealed a different range of wettability. Aligned scaffolds demonstrated increased hydrophobicity compared with randomly oriented scaffolds. The aligned fibers, being compactly packed, yield lower porosity in contrast to the loosely arranged, highly porous random fibers, which influence the wettability.^37^

**Table 3:** Contact Angle of PCL-R, PCL-A, GPCL-R, and GPCL-A Nanofibers

scaffold	left contact angle (°)	right contact angle (°)
PCL-R	134.9 ± 3.1	135.5 ± 3.0
PCL-A	128.4 ± 3.2	127.6 ± 3.0
GPCL-R	77.8 ± 8.0	78.1 ± 3.9
GPCL-A	64.6 ± 8.0	65.1 ± 3.9

### 3.5 Mechanical Testing

The mechanical properties of random and aligned nanofibers with modifications were compared using a universal tensile testing machine. As observed in Figure 3iiA, galactose-modified scaffolds have demonstrated good elasticity along with moderate tensile strength. Random galactose-modified PCL showed the highest tensile strength of 0.041 ± 0.01 MPa when compared to random unmodified PCL with 0.026 ± 0.01 MPa, aligned unmodified PCL with 0.011 ± 0.001 MPa and aligned modified PCL with 0.016 ± 0.002 MPa of tensile strength, respectively. Together, the galactose-conjugated scaffolds, both random and aligned fibers’ tensile strengths were improved when compared to unmodified fibers, indicating that the chemical treatment did not make the scaffolds brittle. Due to random nanofiber orientation, the nanofibers could be drawn out or stretched relatively easily via deformation under the applied stress.^38^

The analysis, as seen in Figure 3iiB, also reveals that random PCL unmodified showed 28 ± 13.3% elongation at break when compared to modified PCL, which shows 49.5 ± 11.6%. Similarly, in aligned unmodified, an elongation at break of 65.3 ± 14.1%, and in aligned modified, 91.3 ± 15.4% elongation at break was observed. In general, materials with higher tensile strength tend to have lower elongation at break, and vice versa. This is because materials that are very strong and rigid (high tensile strength) are less likely to deform or elongate significantly before breaking, whereas materials that are more flexible and ductile (low tensile strength) can stretch and deform more before reaching their breaking point.^39^

### 3.6 Degradation and Water Uptake

Scaffold degradation is essential in tissue engineering as it facilitates the gradual breakdown of the scaffold over time, promoting the growth and regeneration of new tissue.^40^ Under physiological conditions, PCL undergoes degradation via ester bond cleavage through hydrolysis.^41^ Therefore, water uptake influences the degradation and mechanical properties of the polymer.^42^ The aim of this experiment is to compare the water uptake and degradation behavior of electrospun PCL nanofibers with and without surface modification, as degradation rates are influenced by the structure, geometry of the polymers, and surface area.^43^Figure 3iii,iv presents the weight loss percentage and water absorption of electrospun PCL nanofiber surface-modified and unmodified, respectively. Both modified and unmodified scaffolds remained almost unchanged during the degradation period, with both absorption and weight loss remaining very low after 21 days. PCL-R fibers showed 8% uptake at 21 days, while surface-modified scaffolds showed 12% uptake. Similarly, PCL-A fibers showed 7% uptake, whereas surface-modified scaffolds showed 13% uptake, as depicted in Figure 3iii. The higher the water uptake, the higher the degradation that was observed, particularly in GPCL-A. However, there was not much difference in the morphology of fibers observed, as shown in Supporting Figure S1.

### 3.7 MTT Assay

**Figure 4:**
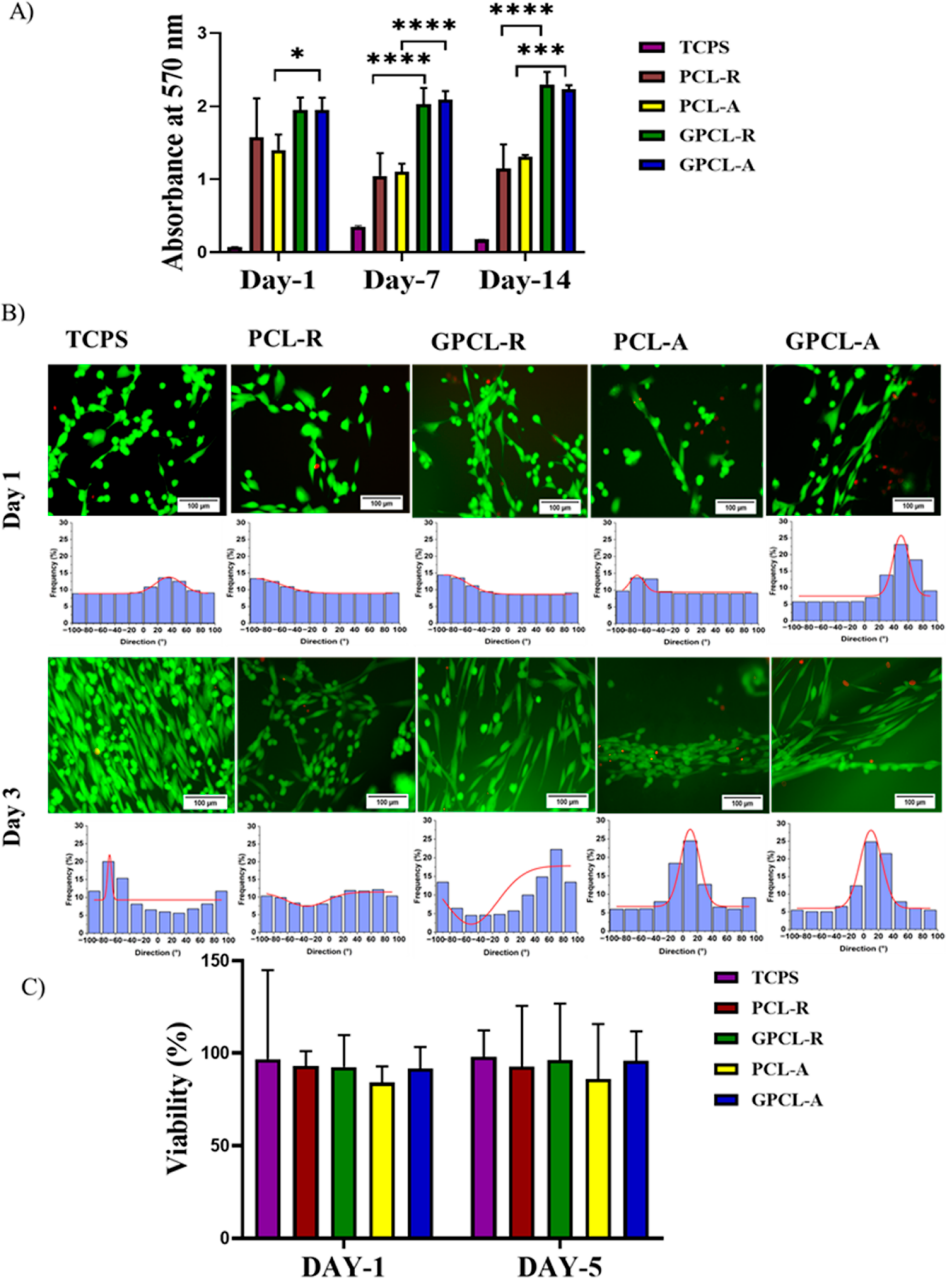
(A) MTT assay using human uterine fibroblasts on TCPS, PCL-R, PCL-A, GPCL-R, and GPCL-A nanofibers on days 1, 7, and 14 of culture (*p < 0.05), (***p < 0.0001). (B) Live/dead assay with human uterine fibroblasts seeded on TCPS, PCL-R, GPCL-R, PCL-A, and GPCL-A scaffolds. (C) Quantification of live cells (green) in live dead assay for viability percentage.

The proliferation of primary HUF on galactose-conjugated nanofibers was evaluated after days 1, 7, and 14 time points. The number of live cells on PCL unmodified and modified was similar at the first day time point. The cell viability on the surface of modified scaffolds increased significantly as compared with unmodified scaffolds after all the time points. In Figure 4A, it is evident that the absorbance of PCL-R and PCL-A on days 7 and 14 is lower than that on day 1. The decrease in absorbance of PCL-R and PCL-A over time may be attributed to several factors, including potential cell confluence, nutrient depletion, or metabolic changes. MTT results also suggest that the modified scaffolds are not toxic to cells and, therefore, are cytocompatible. Moreover, these results imply that enhanced cell proliferation contributes to improved regenerative potential.^22^ While galactose-grafted scaffolds exhibited superior proliferation rates of HUF cells compared with the unmodified PCL scaffolds, this outcome aligns with our expectations. The lack of significant changes in the absorbance of GPCL-A and GPCL-R could be indicative of the enhanced performance of the galactose-grafted scaffolds in maintaining cell viability and supporting prolonged cell proliferation. The interaction between the galactose moiety and cell receptors promoted enhanced cell attachment, resulting in significantly higher proliferation rates at all time points. These findings affirm the preference of HUF cells for a hydrophilic surface.^44^ This is because galactose acts as a cellular matrix adhesive component and triggers the cellular response. Galectin receptors present on fibroblasts are activated, and cell proliferation has increased.^45,46^

### 3.8 Live/Dead Assay

The live-dead test was performed on two-day points, day 1 and day 3. All the scaffolds had very few dead cells, and the difference in morphology on each scaffold could be visualized in Figure 4B. Similar to the TCPS control, most of the cells on the scaffolds were not stressed or dead. It was also noted that the cells seeded on aligned nanofibers exhibited elongation and alignment similar to nanofiber orientation, closely resembling the underlying scaffold morphology. These results in Figure 4B clearly indicate that the topology of the PCL scaffold has the ability to influence the orientation of cell growth and spreading.^47^ On random unmodified and galactose-modified PCL scaffolds, cells tend to spread randomly in all directions. Using the ImageJ software directionality plugin, the degree of direction was calculated, and it was observed that on PCL-A and GPCL-A scaffolds, on day 3, cells are observed to align themselves at an angle of approximately 5 and 16°, respectively. This study suggests that after 3 days of culture, cells exhibit elongation along the direction of the nanofibers, possibly in response to the detected convex curvature of the cylindrical nanofiber structures. Given that aligned fibers mimic the ECM structure of the uterine myometrial layer, they may be a preferred choice over random fibers for certain applications.

### 3.9 Immunofluorescence Assay

Phalloidin staining is used for staining F-actin filaments, which are an important component of the cytoskeleton. The F-actin protein plays a major role in cells as a structural and translocation protein. Numerous signals, such as growth factors, ECM, and chemokines, cause cytoskeletal rearrangement.^48^ In this study, the scaffold properties might have caused a change within the cells and caused the rearrangement of f-actin. Therefore, to study the change in the cytoskeleton, this staining was done.

**Figure 5:**
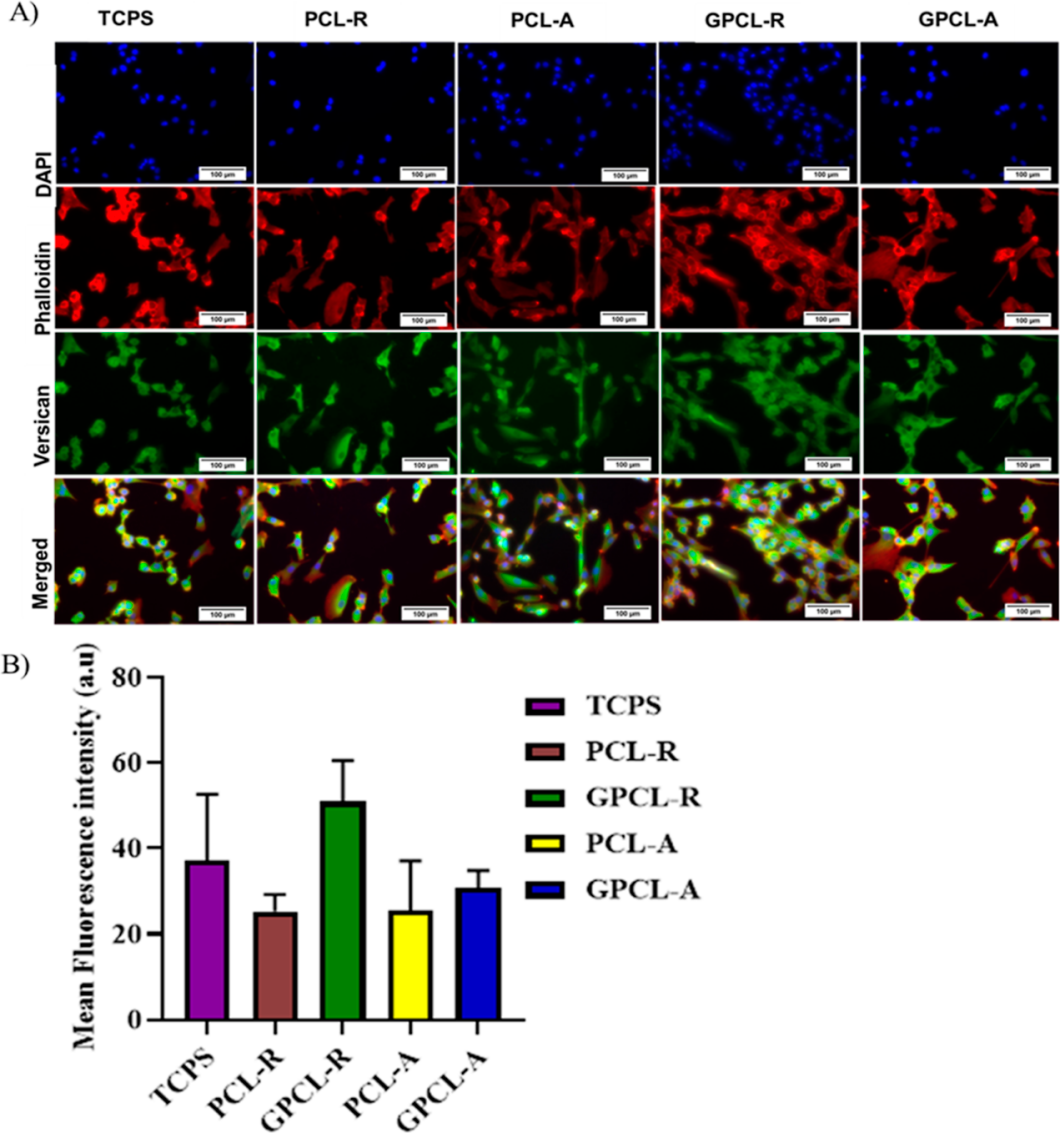
(A) Immunofluorescence assay using DAPI (blue), Rhodamine Phalloidin (red), and Versican (green) for the human uterine fibroblast cells on the modified and unmodified surfaces of the scaffolds after 2 days of culture. Scale bar = 100 μm. (B) Quantification of fluorescence intensity of Versican (green).

To assess the cellular morphology and the arrangement of their actin cytoskeleton on the scaffolds, cells were stained with phalloidin and examined using a fluorescence microscope. The findings, depicted in Figure 5, revealed that the number of cells on unmodified PCL appeared comparatively lower than on the modified counterpart. This observation depicts the impact of the scaffold’s hydrophobic properties on cellular adhesion and distribution. In various recent investigations, it has been shown that cell attachment and spreading are more pronounced on hydrophilic surfaces with positive amine modifications compared to hydrophobic surfaces, under conditions with or without the presence of serum.^49^

Versican is one of the major proteoglycans expressed by cultured fibroblasts and present in the ECM of smooth muscle tissue.^50^ It helps in the binding of hyaluronan via the amino terminal. It also has several other domains to bind to, including lectin, epidermal growth factor, and complement regulatory proteins. The major role of Versican is that it helps in cell adhesion and modulation of the ECM.^51^ To visualize the distribution of Versican in HUFs since it plays a major role in cell phenotype and cell migration, this staining was performed. In Figure 5, it was observed that the morphology and expression of Versican were observed on scaffolds similar to the TCPS. Surface-modified scaffolds exhibited improved expression of Versican by providing amino-terminal groups.

### 3.10 Gene Expression Analysis

**Figure 6:**
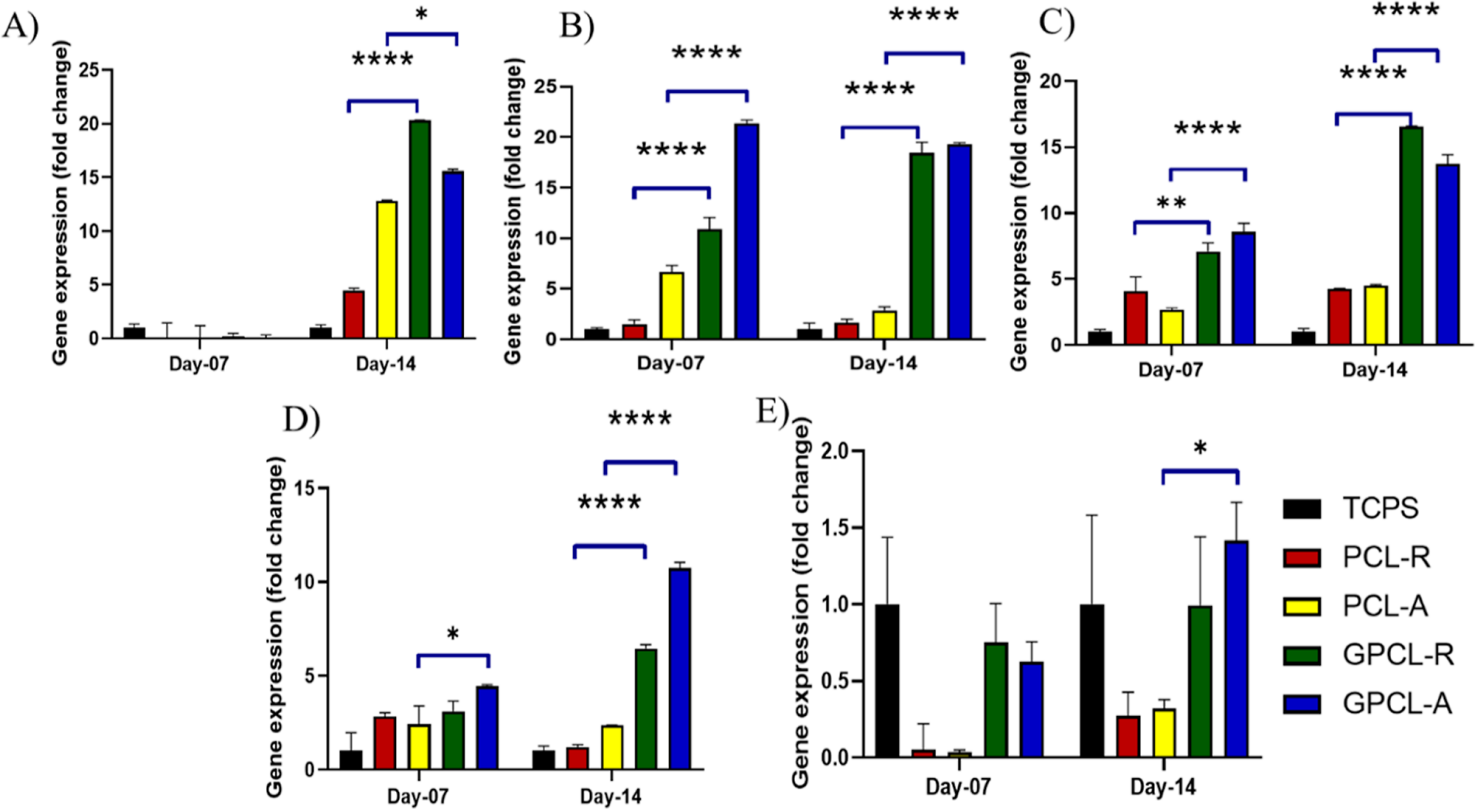
Quantitative real-time RT-PCR gene expression analysis of ECM protein genes. (A) Galectin 3, (B) Versican, (C) laminin, (D) collagen I, and (E) collagen III (*p < 0.05) (****p < 0.0001).

To investigate the impact of galactose conjugation and lectin-based cell–fiber interactions on mimicking trophoblast invasion, cell growth, and potential ECM remodeling, we conducted gene expression experiments. Our hypothesis revolved around the concept of l-selectin-based interactions and potential ECM remodeling. RNA was isolated after 7 and 14 days of culture of HUFs on the scaffolds, and CDNA was prepared. Versican, collagen, laminin, and galectin genes were studied using primers mentioned in Table 1. Galectin 3, a member of the lectin family, possesses unique characteristics for glycan binding. It serves as a versatile regulator of crucial biological processes, including cell adhesion, growth, proliferation, and differentiation.^52^ They also play a role in mediating cell-to-ECM heterotypic adhesion processes. Existing literature suggests that modulating galectin-3 functions can either enhance or diminish cell adhesion to ECM protein ligands like laminin, collagen type IV, and fibronectin.^53^ Additionally, galectin-3 contributes to wound healing and cell re-epithelialization and plays a critical role in modulating interactions between cells and the ECM during wound re-epithelialization.^54,55^ Studies by Bevan et al. confirmed the presence of beta-d-galactoside-binding lectins in the uterine wall, while experiments by Vicovac et al. indicated that galectin-1 and galectin-3 are predominantly found in the placental bed of the uterus, where trophoblast attachment occurs. Galectins also play a role in organizing the ECM and presenting ECM ligands to surface receptors on migrating cells, whether of trophoblastic or bone marrow origin.^56,57^ Our qPCR results in Figure 6A revealed that galectin 3 genes are expressed and upregulated after day 7, peaking at day 14 in galactose-modified scaffolds, particularly in GPCL-R compared to GPCL-A. This upregulation of galectin 3 corresponds to phases of cell proliferation and differentiation. In fibroblast culture, exogenous galectin-3 has been reported to stimulate cell proliferation.^58^ Inohara et al. reported that galectin-3 acts as a mitogen, capable of stimulating fibroblast cell proliferation in a paracrine manner through interactions with cell surface glycoconjugates.^59^

In Figure 6B, Versican, a substantial chondroitin sulfate proteoglycan known for binding hyaluronan and forming extensive ECM aggregates, can influence critical physiological processes such as cell proliferation, adhesion, and migration in the endometrium. It also plays a role in embryo attachment.^60^ We observed significant changes in the expression of Versican on both day 7 and day 14 in galactose-conjugated random and aligned PCL scaffolds. In Figure 6C, laminin, another ECM protein present in the uterus, is typically found in the myometrium and endometrium as part of the basement membranes, particularly in nonpregnant uteri. During embryo attachment and invasion, laminin interacts with trophoblasts.^61^ Therefore, laminin expression is associated with cellular differentiation, adhesion, and growth.^62^ Our study indicated increased laminin expression on day 14 in both modified random and aligned PCL scaffolds, particularly when compared to unmodified scaffolds.^54−57,59^

The ECM of the uterus is characterized as a fiber-reinforced composite viscoelastic material primarily composed of fibrillar collagen, with approximately two-thirds being type I collagen and one-third being type III collagen.^63^ It is important to note that the relative composition of collagen types significantly influences the mechanical properties of the tissues. Maintaining an appropriate balance between collagen types I and III is crucial for preserving the functional integrity of various tissues. An increase in the collagen I/III ratio results in heightened tissue rigidity, whereas a decrease enhances tissue elasticity.^64^ In our study, as shown in Figure 6D,E, we observed significant expression of collagen I in treated scaffolds when compared to collagen III.

In conclusion, our study highlights the profound impact of lectin and galactose conjugation on galactose-conjugated scaffolds. These modifications have shown remarkable improvements in the expression of key ECM components, including galectin, Versican, and laminin. These enhancements in ECM expression signify the potential of these modified scaffolds to mimic trophoblast invasion, promote cell growth, and potentially contribute to ECM remodeling.

### 3.11 In Vivo Biocompatibility of Scaffolds

**Figure 7:**
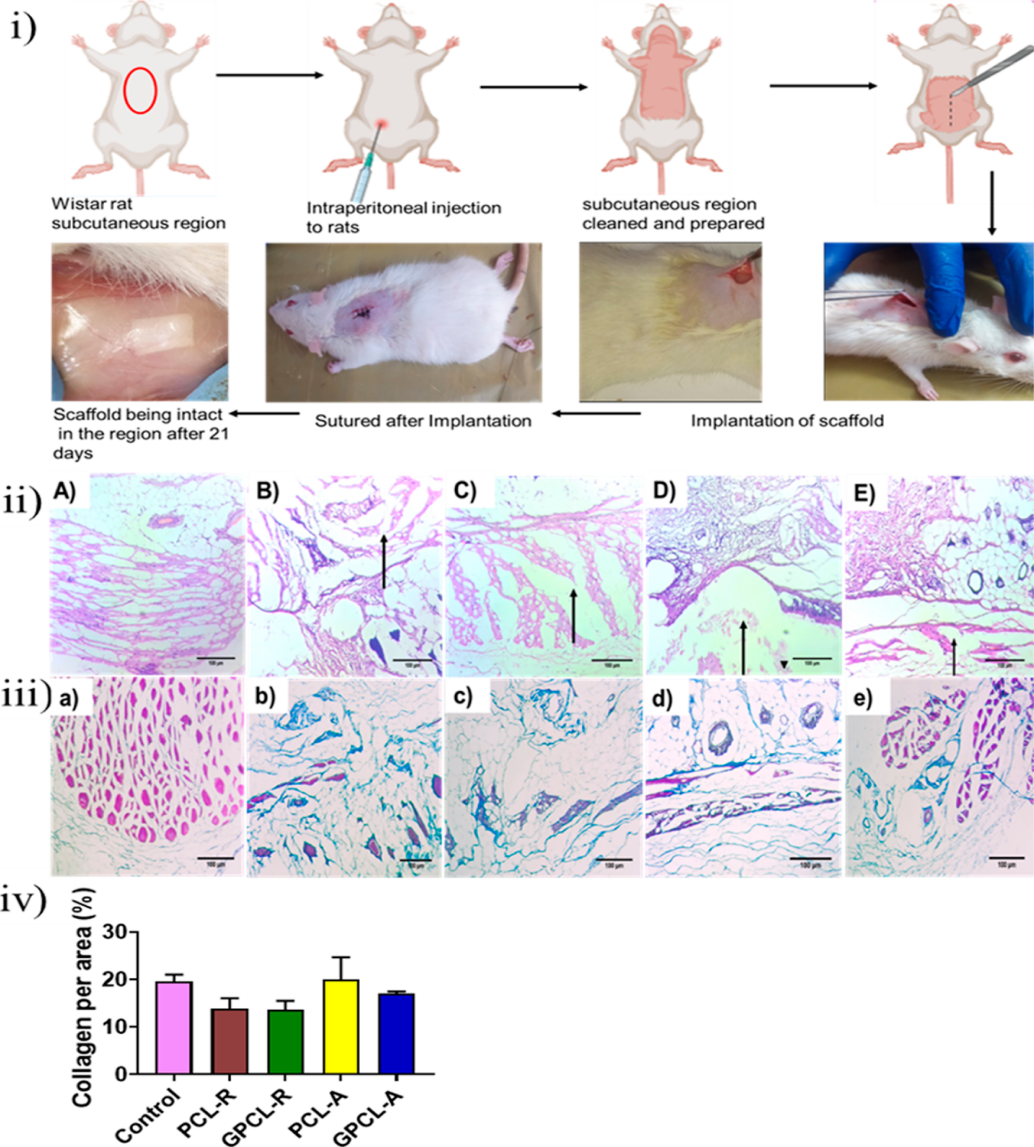
(i) Illustration of steps involved in implanting both modified and unmodified scaffolds in the subcutaneous region of Wistar rats. (ii) Evaluation of tissue integration for the modified and unmodified scaffolds after a 21 day subcutaneous implantation in Wistar rats (*n* = 3) using H&E staining, with the following categories: (A) control, (B) PCL-R, (C) PCL-A, (D) GPCL-R, and (E) GPCL-A. The arrow indicates the muscle layer damage region. (iii) Masson’s trichome staining to assess tissue samples for collagen and scar formation on (A) control, (B) PCL-R, (C) PCL-A, (D) GPCL-R, and (E) GPCL-A. (iv) Quantitative analysis of collagen expression intensity on the scaffolds after Masson’s trichome staining.

Surface-modified nanofibrous scaffolds (1 × 1 cm^2^) were implanted into adult Wistar rats to evaluate their in vivo biocompatibility. Rats (*n* = 3) were subcutaneously implanted with the materials, as shown in Figure 7 and assessed 21 days postimplantation. Continuous monitoring for local inflammation was conducted, including macroscopic and histological evaluations for signs of inflammation and foreign body responses. Throughout the observation period, the rats exhibited normal behavior, and no indications of local inflammation, implant exposure, extrusion, or mortality were observed. Upon macroscopic examination at the time of retrieval, the implants were surrounded by healthy, unaffected tissue devoid of inflammation markers such as redness, swelling, or any adverse tissue reactions that could compromise the integrity of the implanted area.

### 3.12 H&E Staining

H&E staining is a commonly used technique to visualize the morphology of cells and tissues. In the current study, the region of tissue was evaluated, and the degree of damage is classified into three groups: intact, mild, and severe, as shown in Table 4. Table 5 illustrates the degree of damage or intactness in the subcutaneous tissue at the implanted location.^65^ In Figure 7iiB,D, the muscle layer of the PCL-R nanofibers and PCL-R galactose-conjugated nanofibers implanted site showed mild damage. Mild damage to tissue may be characterized by the presence of inflammatory cells, such as neutrophils or lymphocytes, in the affected area. Additionally, there may be changes in the arrangement of cells or the ECM, such as disorganization or increased fibrosis. However, mild damage may not necessarily cause significant changes in tissue morphology or function, and the tissue may still be capable of repair and regeneration. Whereas in Figure 7iiC, PCL-A showed severe damage at the site, which is due to extensive changes in the tissue architecture and cellular morphology. Severe tissue damage may be characterized by the presence of large areas of necrosis, the loss of normal tissue structure, and the infiltration of immune cells, such as neutrophils, macrophages, and lymphocytes. In contrast, PCL-A scaffolds conjugated with galactose Figure 7iiE exhibited significantly less damage, indicating that surface modification mitigates adverse effects and enhances scaffold biocompatibility.

**Table 4:** Histological Parameters

histological parameters	score
tissue	0—intact, 1—focal damage, 2—moderate damage, 3—severe damage

**Table 5:** Score for the Degree of Damage or Intactness in the Biopsy Sections at the Implanted Location

sample	skin	muscle layer	inflammation	subcutaneous fat
control	0	0	0	0
PCL-R	0	1	1	1
PCL-A	0	3	3	2
GPCL-R	0	0	1	0
GPCL-A	0	1	1	0

### 3.13 Masson’s Trichrome Staining

Collagen is a major component of the ECM in connective tissues, including subcutaneous tissue, and is produced by fibroblasts in response to injury or foreign material.^66^ The presence of high levels of collagen in the subcutaneous tissue surrounding the nanofiber implant indicates that the tissue is undergoing a healing response and attempting to isolate the foreign material by forming a fibrous capsule around it. This is a common response to implantable materials and is known as the foreign body response in the first few days after implantation. Whereas in PCL-A unmodified nanofiber samples, a notably higher level of collagen content was observed and quantified from the stained images. The observation of increased collagen content in tissue post implantation of polymer nanofibers suggests that these nanofibers have incited a fibrotic reaction within the tissue, as visually represented in Figure 7iiib,c for the unmodified samples. Additionally, the quantification of collagen intensity revealed higher levels in unmodified PCL-A, as shown in Figure 7iv. While some degree of fibrosis is a normal and expected response to biomaterial implants, excessive fibrosis can impede the integration of the implant with the surrounding tissue, leading to decreased functionality and potential complications.^67^ The modified scaffolds did not cause an excessive degree of fibrosis; therefore, the amount of collagen is similar to the control.

## 4 Conclusions

In conclusion, this study successfully optimized PCL nanofibers via electrospinning, followed by surface modification through a wet chemistry consisting of aminolysis and a galactose conjugation process. The incorporation of sugar groups onto the PCL nanofiber surface was confirmed through a comprehensive ELLA assay, contact angle measurements, and ninhydrin assays. Surface modification enhanced the elasticity of scaffolds, a crucial requirement for myometrium tissue engineering, as demonstrated through mechanical characterization. The biocompatibility of these PCL scaffolds was rigorously assessed through cell culture experiments using HUF cells and subcutaneous implantation in Wistar rats. The results obtained from the MTT assay, live/dead assay, gene expression, and immunofluorescence assays clearly demonstrated the cytocompatibility of the modified PCL scaffolds in terms of cell adhesion, proliferation, and viability. Moreover, these scaffolds exhibited a remarkable enhancement in HUF proliferation compared with pristine PCL scaffolds, signifying their potential for fostering cell growth and tissue regeneration. Additionally, the galactose-conjugated PCL scaffolds induced superior cytoskeletal morphology and upregulated fibroblast ECM marker expression compared to their unmodified counterparts. These findings strongly support the suitability of galactose-conjugated PCL fibers as a versatile platform which activates HUFs to upregulate the regeneration process in myometrium. In vivo subcutaneous implantation results showed that the degree of inflammation and damage in the muscle layer was less in modified scaffolds when compared to unmodified scaffolds. Future investigations could explore the feasibility of using these modified scaffolds with human uterine smooth muscle cells and could provide valuable insights about behavior and the potential for supporting the repair of the uterine myometrium. Additionally, in vivo animal studies in a uterine myometrial injury model will provide better understanding of scaffold’s efficiency and integration in complex host tissues. Insights from these studies could lay the foundation for clinical applications aimed at reducing uterine scarring after C-sections or fibroid surgeries, addressing a significant clinical concern and offering a potential solution for improving patient outcomes in reproductive health. In this study, while both random and aligned PCL fibers were studied, PCL-A fibers conjugated with galactose were preferred over random galactose conjugated fibers because they exhibited significantly less damage in animal studies when compared to unmodified fibers. Also, due to their better mimicry with the uterine myometrial layer, it makes them more advantageous for myometrial tissue engineering applications. In summary, the successful surface modification of PCL nanofibers with galactose conjugation holds immense promise for the development of advanced scaffolds in uterine tissue engineering. This innovative approach opens new horizons for the integration of bioactive components, potentially revolutionizing the field of regenerative medicine for uterine repair and beyond.

## Supporting Information Available

Detailed results from degradation and water uptake studies—representative SEM images illustrating the degradation analysis of the scaffolds (PDF)
