# Supplementary material for: Surface-Conjugated Galactose on Electrospun Polycaprolactone Nanofibers: An Innovative Scaffold for Uterine Tissue Engineering
Source: ACS Omega. 2024 Aug 1;9(32):34314–28. doi: 10.1021/acsomega.3c10445 (PMC11325431; doi:10.1021/acsomega.3c10445)
Supplement: Supplementary file 1 — ao3c10445_si_001.pdf [file ao3c10445_si_001.pdf]

Surface-Conjugated Galactose on Electrospun Polycaprolactone Nanofibers: An Innovative Scaffold for Uterine Tissue Engineering

Srividya Hanuman[1], Harish Kumar B[2], K Sreedhara Ranganath Pai[2], Manasa Nune[1]*

[1]Manipal Institute of Regenerative Medicine, Bengaluru, Manipal Academy of Higher Education, Manipal, Karnataka 576104, India

[2]Department of Pharmacology, Manipal College of Pharmaceutical Sciences, Manipal Academy of Higher Education, Manipal, Karnataka 576104, India

*Corresponding Author email address: manasa.nune@manipal.edu

Supporting Information

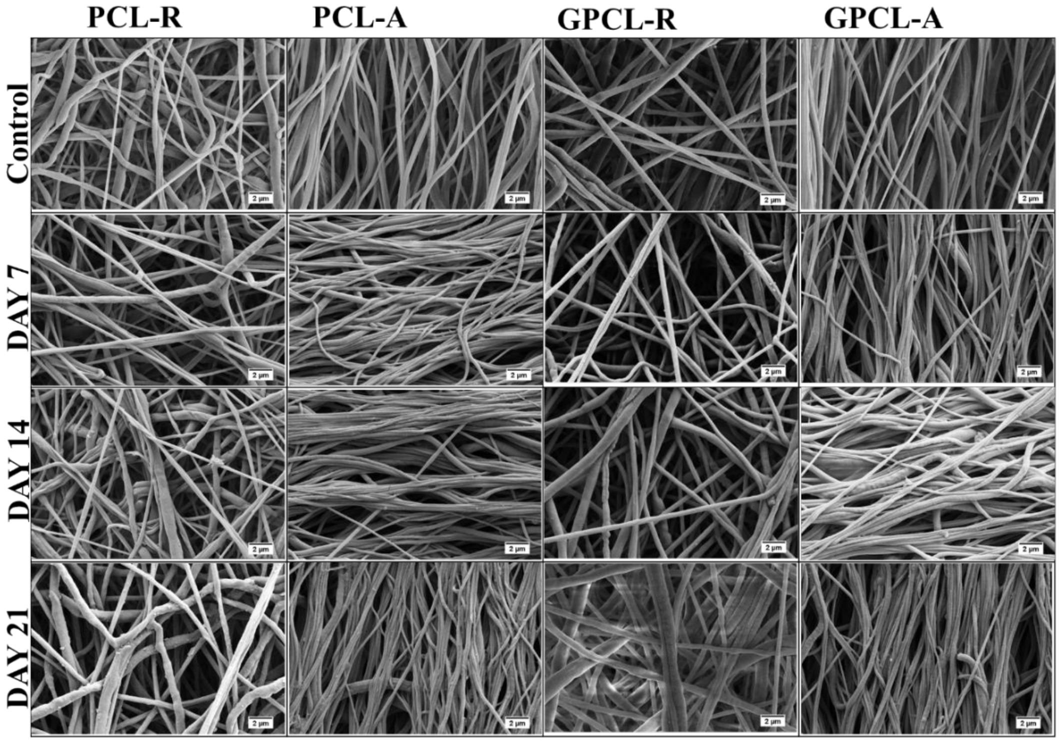

Figure S1. Representative SEM images of degradation analysis of the scaffolds. The first row illustrates the control morphology before degradation of PCL-R, PCL-A, GPCL-R and GPCL-A. Subsequent rows depict the degradation of PCL electrospun meshes at 7, 14, and 21-day time points, Scale bar = 2 µm
